# Delivery systems of CRISPR/Cas9-based cancer gene therapy

**DOI:** 10.1186/s13036-018-0127-2

**Published:** 2018-12-18

**Authors:** Alessio Biagioni, Anna Laurenzana, Francesca Margheri, Anastasia Chillà, Gabriella Fibbi, Mario so

**Affiliations:** Department of Experimental and Clinical Biomedical Sciences, University of Florence, Viale G.B. Morgagni 50 –, 50134 Florence, Italy

**Keywords:** CRISPR, Delivery systems, Gene-editing, Cancer, Gene therapy

## Abstract

CRISPR/Cas9 (Clustered Regularly Interspaced Short Palindromic Repeats) is today one of the most reliable method for gene-editing, supporting previous gene therapies technologies such as TALEN, Meganucleases and ZFNs. There is a growing up number of manuscripts reporting several successful gene-edited cancer cell lines, but the real challenge is to translate this technique to the clinical practice. While treatments for diseases based on a single gene mutation is closer, being possible to target and repair the mutant allele in a selective way generating specific guide RNAs (gRNAs), many steps need to be done to apply CRISPR to face cancer. In this review, we want to give a general overview to the recent advancements in the delivery systems of the CRISPR/Cas9 machinery in cancer therapy.

## Introduction

**Fig. 1:**
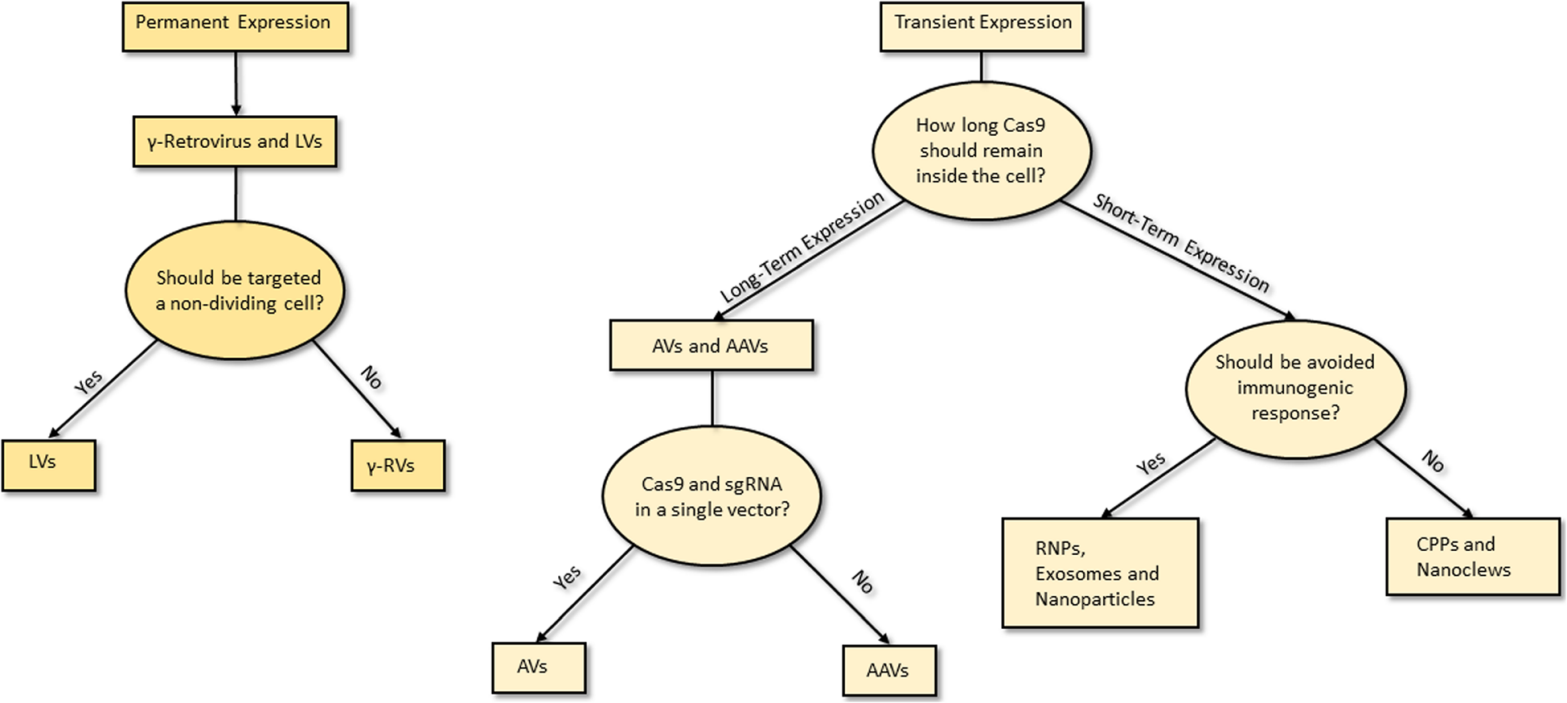
How to choose the best delivery system. A schematic representation on the main topics that should be dealt with to choose the best carrier for CRISPR machinery

Cancer, unlike other genetic diseases such as Duchenne Muscle Dystrophy, HPV, HBV, Cystic Fibrosis, etc., relies on several genetic mutations. Indeed, it is widely known that dysregulation of not a single gene, but multiple genes leads to cancer. Therefore, the first issue for cancer gene therapy is that editing a single gene is often not sufficient. From these considerations, it is very important to understand the role of every single mutation that accumulate during cancer progression, in particular for those genes whose alterations play crucial roles in metastasis. Moreover, to complicate a yet hard to understand disease, being multifactorial and multigenic derived, many mutations occur stepwise during progression from early stage tumors to late ones [1]. The pool of cells composing the tumor bulk presents several genetic alterations but during tumor evolution, the gatekeeper mutations provide a selective growth advantage to some clones that acquire the capacity to resist therapies and keep growing, thus overwhelming the surrounding cells [2–4]. In order to restore the sensitivity to chemo- and radiotherapies the genes responsible for resistance should be corrected. Indeed, several studies are currently ongoing on the use of CRISPR to knockout gain-of-function tumor mutations [5]. CRISPR (Clustered Regularly Interspaced Short Palindromic Repeats) is a prokaryotic adaptable immune mechanism exploited by bacteria and archaea to protect themselves from foreign nucleic acids. This complex system, which has been adapted to be used in laboratory practice, can recognize and cut DNA to provide a complete and high selective gene editing in vitro and in vivo. The possibility to be used in clinical treatment for several genetic derived pathologies has rapidly spread its fame worldwide [6]. Another obstacle in the race for the perfect gene therapy is that, the therapeutic translation of the CRISPR/Cas9 system lacks an appropriate delivery carrier [7]. Even when a specific molecular target is available to select tumor cells, it is quite hard to identify an accurate transport system, which may contain all the machinery. To overcome this issue, researchers exploited several kinds of carriers, from viral delivery system to cation lipids, from nanoparticles to nanomolecular DNA traps, each one with pro and cons to be considered accurately (Fig. 1). In this review, we will consider the most reliable and used delivery methods worldwide but being the CRISPR-based therapies still at the beginning of their development, many aspects need to be further investigated in the future.

### Modes of DNA editing and repair

To face cancer as a multiple gene-based disease, CRISPR needs to knockout or repair mutant alleles that are responsible for the tumor malignancy. Cas9 could induce double strand breaks (DSBs) which are repaired exploiting the Non Homologous End Join (NHEJ) repair system, mediating indel mutations formation and producing a frameshift effect within the coding region of a gene, thus generating a premature stop codon which is responsible for the knockout of the target gene expression. While NHEJ is an error-prone repair pathway, simply rejoining the ends of the DSBs, in case a single-stranded oligonucleotide is provided, acting as a donor template, the cell will repair the created nick by Homologous Directed Repair (HDR) editing in this way the DNA [8]. Moreover, introducing D10A or H840A mutation into the RuvC- or HNH-like domains of the spCas9, the nuclease will be unable to perform a DSB, generating only a single cut per strand. These mutants also known as Nickase are extremely useful to improve the specificity of the cut, albeit with a reduced efficiency, using two gRNA instead of only one. Indeed, DNA single-strand breaks are repaired via the high-fidelity base excision repair (BER) pathway without performing any editing of the target gene [9]. The most common and easiest approach to CRISPR-mediated cancer treatment is the gene knockout exploiting the NHEJ. In such a way, the nuclease should be directed towards genes involved in chemoresistance, proliferation, migration, invasion and apoptosis resistance in order to inhibit metastasis and tumor growth. Obviously the limiting step is the selectivity of the method. Being all these target genes ubiquitously expressed, a selective delivery method to transfer the CRISPR machinery only in cancer cells is required. When the target is a mutation, the selective method is to exploit HDR instead. Indeed, providing a homologous template it is possible to restore the mutant gene to a wild-type genotype thus inhibiting the biological effect of such mutation, both in the promoter region or in the coding region of the target gene. We do believe that both strategies are applicable to cancer treatment depending on the case but, generally, exons are preferentially targeted despite of promoters, enhancers or TATA box which might be long non coding region often not completely known. Finally, CRISPR could also be used to prime immune cells, such as T cells, enhancing their anti-tumor activity by abrogating the expression of PD-1, DGK and FASL, especially in combination with expression of chimeric antigen receptor (CAR) in T-cell immune therapies [10–13]. Such T cells, are collected from patients by leukapheresis and modified in vitro to express CAR gene using viral or non-viral vectors. Thus, the knockout of the above listed genes involved in the main check points of the immune cells regulation, may fuel T cell response enhancing their anti-tumor activity once reinfused in the patients.

### Adenoviruses and adeno-associated viruses

Among viruses, the most widely used are Adenoviruses (AVs) and Adeno-Associated Viruses (AAVs) due to their high transfection efficiency and the high-titers of production [14]. Another advantage of AAVs is that they can be transduced in both dividing and non-dividing cells and do not generally integrate into the host genome [15]. Indeed, for many applications, transient expression of gRNAs and Cas9 is typically sufficient to induce efficient genome editing. Therefore, after expression and selection, plasmid expressing CRISPR machinery is usually lost, avoiding that extended persistence in the cell could lead to increased frequencies of off-target mutations [16]. Moreover, AVs are widely used for cancer therapy due to their ability to preferentially infect cancer cells, exploited in the commonly called oncolytic therapy [17]. Plasmids keep part of their viral parental structure while gRNA and Cas9 could be easily cloned and packed into viral particles, which can be commonly delivered via intramuscular or intraperitoneal injection. Maggio I et al. demonstrated that AVs delivery of the CRISPR machinery should be optimized for the use in cancer. Indeed, they tested on several cancer and non-cancer cell lines, such as HeLa, U2OS, hMSCs and myoblasts, the optimal ratio of AVs encoding Cas9 and gRNA, confirming that, integrated vector designs in which both Cas9 and gRNA expression are co-delivered within single vector particles, are advantageous in terms of knockout efficiency [18]. This phenomenon should be taken into consideration designing the plasmid due to the relatively large size of Cas9 ORF that may compromise the production of viral vectors being the packaging capacity offered by AAVs significantly lower than AVs. To avoid such issue, it could be used the Cas9 derived from *Staphylococcus aureus* (SaCas9) which is ~ 1 kb shorter than the classical SpCas9 and can edit the mammalian genome in vivo as well, even if leaded by an alternative protospacer-adjacent motif (PAM) sequence [19]. Taeyoung K et al. targeted in non-small cell lung cancer a mutant version of EGFR harboring a single-nucleotide missense mutation that generates a PAM sequence recognized by the common Cas9 derived from Streptococcus pyogenes. They co-delivered Cas9 and the EGFR mutation-specific gRNA exploiting adenoviruses as delivery system via intra-tumor injection and the result was a precise disruption of the oncogenic mutated allele with high specificity [20].

### Retroviruses

When the expression must be permanent, so Cas9 and/or gRNA must be integrated into the hosting genome, the best choice is to use retroviruses. Among them, the most used ones are γ-retroviruses and lentiviruses (LVs). All the members of such big family share the reverse transcriptase being their genome composed by RNA instead of DNA. γ-retroviruses are the least used because they can only transduce dividing cells being able to enter the nucleus during the mitotic breakdown of the nuclear envelope [21]. Additionally, γ-retroviruses as well as LVs integrate randomly into the host genome, being potentially mutagenic and oncogenic. The last ones are commonly derived by HIV-1 thus requiring specific handling procedure and protocols although the so called third generation lentiviral system has been designed to be safer for researchers. Indeed, this system is based on four plasmids to be used for lentiviral particles formation and packaging: one plasmid encodes for the Envelope protein, one for Gag and Pol (structural proteins), one for Rev. (transactivating protein) and the last one contains the gene to be expressed [22]. Annunziato S et al. reported an intriguing effect of CRISPR machinery delivered via lentiviruses: they described an innovative approach to model invasive lobular breast carcinoma by intraductal injection of lentiviral vectors encoding Cre recombinase, the CRISPR/Cas9 system, or both, in female mice carrying conditional alleles of the Cdh1 gene, encoding for E-Cadherin. Pten Cre-mediated editing in mice with mammary gland-specific loss of E-cadherin efficiently generates carcinoma-initiating cell that developed intraductal cell carcinoma while the infection with the Cas9 system stimulated an immune response that limited the success of Pten knockout inducing tumor that do not resemble the intraductal histotype [23]. Indeed, Cas9 was already reported to cause immunogenic response [24]. Another recent study involving gene editing mediated by lentiviral delivery was made by Zhao G et al. reporting that BIRC5 gene KO inhibited the Epithelial to Mesenchymal Transition (EMT) in ovarian cancer cells by upregulating epithelial cell markers such as cytokeratin 7 and downregulating mesenchymal markers like Snail2, β-Catenin, and Vimentin while overexpression of BIRC5 promoted EMT [25]. Huo W et al. also reported the use of CRISPR machinery delivered via lentivirus for the knockout of miR-21 [26], an onco-miRNA that is commonly upregulated in cancer and promotes tumor metastasis and chemoresistance. Inducing a mutation in the pre-miRNA sequence, they caused the complete loss of miR-21 expression and consequent reduction of cell proliferation, migration and invasion in two ovarian cancer cell lines. In the end, they demonstrated that abrogation of miR-21 inhibited the EMT upregulating E-Cadherin and downregulating Vimentin and Slug.

### Fusion proteins of Cas9

Among the most used non-viral system to delivery CRISPR machinery there is the use of the exogenous form of the spCas9 mixed with the targeting gene gRNA introduced into cells by lipid-mediated delivery [27]. In this way, eukaryotic cells only undergo the gene knockout without the need to synthetize the gRNA and the Cas9. Editing by the use of combined ribonucleoproteins (RNPs) Cas9-gRNA presents many advantages including a very rapid and robust knockout process and the complete clearing of Cas9 after 24 h from the transfection, decreasing off-target effects [28]. Moreover, compared to plasmid transfection, RNPs demonstrated reduced off-target mutagenesis and cell death and avoid also the risk of insertional mutagenesis by integration of the vector into the host genome [29–31]. RNPs could also be used to edit fertilized eggs by direct mechanical injection and this has been applied to zebrafish, rats and mice [32–34]. An interesting application of CRISPR was reported by Levi J. Rupp et al. that demonstrated improved therapeutic efficacy of Cas9-edited CAR-T cells and highlighted that this new methodology may enhance next-generation cell therapies. Indeed, they developed a protocol for combined Cas9 RNPs-mediated gene editing and lentiviral transduction to generate PD-1 deficient anti-CD19 CAR-T cells. In this way, PD-1 disruption augmented CAR-T cell mediated killing of tumor cells in vitro and enhanced clearance of PD-L1+ tumor xenografts in vivo [35]. Moreover, as reported by Sun L et al. RNPs do not activate the cyclic GMP-AMP synthase, which is the cytosolic signal to trigger the immune response [36]. Another simple and fascinating method to generate enhanced Cas9 is the cell penetrating peptides (CPPs)-based technology [37]. First discovered in the antennapedia homeodomain and the HIV-1 TAT protein [38, 39], CPPs are short peptide sequences that can easily pass through cellular membranes and deliver CRISPR machinery. Indeed, once conjugated the Cas9 to CPPs through a thioether bond and the gRNA complexed with CPPs, forming positively charged nanoparticles, they can be used to treat cells directly without the need to use any additional transfection reagents. In this way, Ramakrishna S et al. conjugating Cas9 protein with 4-maleimidobutyryl-GGGRRRRRRRRRLLLL and a gRNA to the C3G9R4LC peptide, abrogated efficiently and without any off-target effects, CCR5 gene in embryonic stem cells, dermal fibroblasts, HEK293T cells, HeLa cells, and embryonic carcinoma cells [40].

### Membrane-derived vesicles

CRISPR is an excellent tool especially for therapeutic purposes for all the genetic diseases and for cancer but its use in vivo is limited to date due to the immunogenic response that certain kind of carriers might cause. To overcome this issue, Seung MK et al. reported the use of cancer-derived exosomes [41]. Indeed, exosomes are nanospherical membrane-type structures with a bilayer of lipids [42] quite similar to the cellular membrane, ranging from 30 nm to 120 nm in diameter, known to originate by budding from the internal vesicles of multivesicular bodies and released into the extracellular milieu. They are commonly produced and secreted by numerous cell types, including immune, epithelial, endothelial and tumor cells. Recently it was discovered, through a deep proteomic and transcriptomic analysis, that exosomes may contain several proteins, mRNAs, long non-coding RNAs and miRNA [43, 44]. Thus, their ability to carry various molecules was already exploited in vivo thanks to their low immunogenicity [45, 46]. Moreover, the cancer-derived kind of exosomes offer the particular capacity to accumulate in tumors. In this way, Seung MK et al. suppressed the expression of poly (ADP-ribose) polymerase-1 both in vitro and in vivo, inducing apoptosis in the ovarian cancer line SKOV3, enhancing also the chemo-sensitivity to cisplatin. However, their use is commonly limited by their low efficiency in the encapsulation of large nucleic acids [47].

### Nanoformulations

Mout R et al. reported an alternative editing strategy using gold nanoparticles to generate nanoassemblies composed by an engineered form of the Cas9 protein and a gRNA [48]. Indeed, Cas9 was modified inserting a glutamate peptide tag to the N-terminus, to self-assembly with cationic arginine gold nanoparticles (ArgNPs). Moreover, to increase the efficiency of nuclear transport they added also a nuclear localization signal (NLS) to the C-terminus. This method resulted to be very useful to deliver proteins and nucleic acids into the cytoplasm and to obtain an efficient transport to the nucleus, especially if paired with the innovative use of gold nanoparticles for therapeutic use [49, 50]. Nanoparticles may be also generated with a core formed by polyethylenimine (PEI) hydrogel for the encapsulation of Cas9 protein while the external shell is made of cationic 1,2-dioleoyl-3-trimethylammonium-propane chloride salt (DOTAP) lipids, required for the delivery of genetic materials. These hybrid nanoparticles called liposome-templated hydrogel nanoparticles (LHNPs) were designed for the first time by Chen Z et al. [51] to have the ability to selectively reach the brain thanks to an autocatalytic tumor-targeting poly(amine-co-ester) terpolymer and to penetrate easily the blood-brain barrier [52]. Another interesting application is the use of Cr-Nanocomplex, where recombinant Cas9 was covalently modified with branched polyethylenimine as the carrier for packaging sgRNA, enhancing the delivery efficiency into methicillin-resistant *Staphylococcus aureus* [53]. The last and newest delivery system reported in this review is called Nanoclews, which are yarn-like single strand DNA nanoparticles synthesized by rolling circle amplification [54]. Such nanomolecular traps could encapsulate chemotherapeutic agents controlling their release, depending on the microenvironmental conditions [55]. These kind of nano-objects have been exploited to load the Cas9 protein and the gRNA and coated with the cationic polymer polyethylenimine to induce endosomal escape. After cell absorption by endocytosis, nanoclews deliver their cargo into the cellular nuclei thanks to a NLS presents on the Cas9. However, being a new and unexplored technology, it should be further investigated before being translated in the clinical practice due to the potential immunogenicity effects in vivo.

### Genetically engineered mouse models

To better understand the cancer genetic profile, the mechanisms for metastasis and chemoresistance and to discover new biomarkers there is a growing need to develop accurate and reproducible mouse models. Both in basic and in translational research the most common models used are the cancer cell line transplantation, where a stabilized line of human or murine cancer is inoculated and developed in mice, and patient-derived tumor xenografts where fresh biopsies are collected from patients and then transplanted in mice [56]. Obviously only immunocompromised mice should be employed to avoid xenograft and allogenic rejection, losing an important biological element, such as the function of the host immune system. Even if used since 50 years worldwide, these murine models are rapidly replaced by genetically engineered mouse models (GEMMs) using CRISPR/Cas9 technology [57]. Exploiting this gene editing mechanism we are able to introduce every genetic alteration, present in human, both in murine embryos or in adult mice, using immunodeficient mice or animals that shown immunological tolerance to Cas9. Indeed, murine embryonic stem cells can be genetically modified to bear human mutations and once injected in a blastocyst, generate a chimeric mice [58]. CRISPR may also be used to edit murine genome by local administration of lentiviruses encoding a target sgRNA in transgenic mice with tissue-specific Cas9 expression or by lentiviruses encoding both Cas9 and sgRNA in wild-type mice. Such animal model can be successfully exploited to validate new oncogenes, to identify the mutation needed for cancer initiation, to study the relationship between tumor cells and the microenvironment and to identify new drugs. However, there are several limitations to their use such as the low incidence of metastatic spread and the different organ tropism respect to humans [59]. Another issue using GEMMs is the latency between tumor burden and the development of metastatic lesions [60]. Moreover, further developments need to be done in order to reduce time and cost for GEMMs.

## Discussion

To date, gene therapy and CRISPR represent not only the hopes for many patients affected by monogenic diseases, such as Duchenne Muscle Dystrophy [61], Cystic Fibrosis [62], familial hypercholesterolemia [63] and viral infections, like HPV [64], HBV [65], HIV [66] but also the possibility to improve the life quality of many people fighting against multifactorial syndromes such as diabetes [67], Alzheimer [68] and Parkinson [69]. However, while it is feasible to study and edit a single mutation, it is difficult to restore the correct expression of different mutated genes like in cancer. We do believe that even if the genetic profile of many tumors is well known and many mutations are recurrent depending on the tumor tissue, personalized medicine development may fuel the knowledge of CRISPR targets. Thus, cancer gene therapy based on CRISPR use might strike selective mutations or genes important for the tumor survival, combining this genetic therapy with the use of chemotherapies in order to hit the tumor with different approaches at the same time. Moreover, cancer does not offer a specific and universal molecular target to direct selectively a therapy to tumor cell only avoiding side effects on healthy cells. The most important marker commonly reported is HER2, that is overexpressed in some subset cases of breast, ovarian, gastric, colorectal, pancreatic and endometrial cancers, targeted by Trastuzumab [70], but in the majority of cases the lack of specific markers make promising target therapies non-exploitable. CRISPR based gene therapy offer the advantage that a specific designed gRNA could target precisely only one mutation. Therefore, in case CRISPR machinery is delivered into a non-mutated cell, at least theoretically, it does not perform any knockout or editing and its components should be rapidly removed. On the other hand, this gene-editing platform is not completely error proof and in particular, when gRNAs are not specific and Cas9 is expressed at high levels, it could lead to off-target effects [71]. The delivery system plays a crucial role as well, enabling the CRISPR machinery to reach all the mutated cells, avoiding mosaicism effects due to low loading and releasing capacity [72]. There are several delivery methods used in vivo and in vitro currently evaluated for their applicability in human gene therapy. Hydrodynamic injection can deliver large macromolecules such as RNPs in vivo by injecting a solution intravenously at extremely high volume and pressure thus causing the temporary opening of pores in the vasculature through which molecules enter penetrating into several tissues. While this method has successfully allowed to deliver plasmids encoding Cas9 and gRNA into the heart, lungs, liver, and kidney tissue [73] of mice, its use is restricted to small animal models due to the large injection volume, making it not currently appropriate for human application. Electroporation is another transfection methods recently highly optimized for genome editing used in ex vivo cell therapy, such as reported above for CAR-T cells reprogramming, but currently not feasible for in vivo clinical use [74]. Virus represents one of the most intriguing ways to efficiently deliver the content of the CRISPR machinery in tumor cells. In particular, AVs and AAVs are exploited thanks to their capacity to transport plasmids encoding the gRNA and the Cas9 with a short-term expression. Indeed, LVs are usually less used due to their ability to integrate into the host genome possibly causing oncogenic and/or heritable mutations. One possible alternative to canonical LVs is to exploit the mutations of the integrase viral enzyme that prevents proviral integration resulting in increasing the expression levels of circular vector episomes in infected cells [75]. Therefore, these integration-deficient lentiviral vectors (IDLVs) are gradually lost by dilution in dividing cells and are stable for longer period in quiescent cells. IDLVs, compared to wild-type LVs, have a significantly reduced risk of causing insertional oncogenic mutations and the transient expression of CRISPR machinery decreases also the risk of off-target effects (Table 1). Moreover, to increase the efficiency and the specificity of viral-mediated gene-editing delivery, viruses can be mixed with other viruses’ parts, creating new hybrids, or fused with small molecules such as synthetic polymers and inorganic nanoparticles [76]. However, the most remarkable breakthrough in the last few years is the use of RNPs. The exogenous form of Cas9 complexed with the target gRNA confers great specificity and efficiency without the issue related to the immunogenic response. Moreover, RNPs may be transfected into cells both via electroporation and lipofection for the in vitro and via conjugated liposomes for the in vivo practice. Particularly interesting is the combination of liposomes with exosomes reported by Li et al. creating a new hybrids [77]. This simple system, encapsulating CRISPR machinery via an incubation with exosomes and liposomes, makes the resultant hybrid nanoparticles easily endocytosed by hard to transfect cells and it does not give rise to immunogenic response. ArgNPs were also reported to be one fascinating and extremely efficient method that could be coupled with the use of gold nanoparticles for therapeutic practice. Indeed, gold nanoparticles demonstrated a great potential in the ablation of solid tumors [49, 50], an innovative approach that could be potentiated by CRISPR-mediated gene therapy. There are many other new technologies to deliver CRISPR/Cas9 such as CPPs and Nanoclews but with the evident limit posed by the strong immunogenic response reported in the in vivo experiments. Moreover, while in monogenic diseases is often sufficient to edit a limited number of cells to observe a biological effect, in cancer gene therapy the great issue is to target the population of cancer cells, which have a growth advantage over the healthy tissues, diluting quickly the number of edited cells and thus making the treatment ineffective. As a result, repeated injection are needed and higher editing efficiencies are strongly required to be therapeutic, which is challenging for the current CRISPR technologies [78].

**Table 1:** Pros and Cons analysis of the delivery methods. na: not applicable

System	Pros	Cons	Packaging Size	Immunogenicity	Insertional Mutagenesis	Tissue/Cell Tropism	Ref.
AVs	High packing capacity	High immunogenic response	> 8 Kb	High	No	Yes	[14–20]
AAVs	Low immunogenic response, small viral particle size	Low packing capacity	⁓4.5 Kb	Tissue dependent	No	Yes	[14–20]
γ-Retroviruses	High packing capacity	Only dividing cells can be infected, genome integration of target sequence and high risk of oncogenic mutations	< 8 Kb	Moderate	Yes	Yes	[21]
LVs	Low immunogenic response	Genome integration of target sequence and high risk of oncogenic mutations	< 8 Kb	Low	Yes	No	[22–26]
RNPs	Low immunogenic response	Cells cannot be selected with antibiotics or fluorescent markers	na	Low	No	No	[27–36]
CPPs	No transfection reagents need to be used	Cas9 needs to be chemically conjugated to CPPs	na	CPP dependent	No	No	[37–40]
Exosomes	Low immunogenic response, Self-accumulating in tumor mass	Low efficiency of encapsulation, easily degraded	Exosome size dependent	Low	No	Yes	[41–47]
Nanoparticles	Can be conjugated with chemical or physical compounds	Difficult to use	na	Nanoparticle dependent	No	Nanoparticle dependent	[48–53]
Nanoclews	Release dependent on the microenvironment conditions	High immunogenic response	na	High	No	Nanoclew dependent	[54, 55]
IDLVs	Reduced risk of insertional oncogenic mutations	Genome integration of target sequence in non-dividing cells	< 8 Kb	Low	Only in non-dividing cells	No	[70]

### Concluding remarks

In last, we can affirm that while gene therapy has found an innovative and extremely efficient tool in CRISPR/Cas9, many efforts need to be spent to find not only a specific molecular target for cancer, but also a selective delivery method. Indeed, many of the above described systems present several pitfalls that should be overstep in order to translate their use in clinical practice. For many years, the majority of the approved gene therapies was based on the use of AAVs and AVs while to date scientific community is developing nanoparticles-like structure that could be easily loaded with the gene-editing machinery, as plasmid or RNPs, and with low cytotoxic and immunogenic effects. Among the described delivery systems, we encourage the use of AVs and AAVs to study the molecular effects of in vitro gene editing. Indeed, the high efficiency and the possibility to positively select transfected cells via specific markers of selection allow generating pure edited cell lines from single cloned cells, generating a genetically homogenous population. Although this system is the most affordable and easy to be used in vitro, it is not totally feasible for cancer treatment. Moreover, human immune response to gene therapy may vary significantly depending on the tissue site of injection, with outcomes ranging from unresponsiveness (gene transfer in the eye, i.e. Luxturna for RPE65 mutation), to tolerance, to clearance of transduced cells. Conversely, RNPs could be used as well but with the disadvantages that currently it is not available a selection markers as for plasmids. Thus, this method should be privileged when testing in vitro new molecular targets to be translated in in vivo applications. In last, among the most promising approaches, several fascinating gene therapies based on the use of chimeric viruses are also in development, which will let to generate high selective and specific organotropic lentiviral particles.

### Future perspective

We do believe that in future the attention of the worldwide research should be pointed to develop new cell-based delivery systems for the human gene therapy. These approaches based on the use of autologous cells derived from patients will let us to exploit their natural homing capacities and to set to zero all cytotoxic and immunogenic effects. The so obtained cells could be easily edited in vitro to be used as shuttle for the CRISPR machinery, which may be transferred to cancer cells via the fusion of the cellular membranes, exploiting specific fusion protein like Sendai virus-derived Protein F and Hemagglutinin-Neuraminidase [79, 80]. Indeed, it is widely known to date that Sendai, exposing the above described proteins on the surface of the infected cell membrane, can generate syncytia. Thus, expressing the two fusion protein under control of an inducible promoter it may be possible to transduce a genome editing machinery, such CRISPR, among cells. Engineering autologous cells collected from patients, that retain a natural tropism for tumors, such as endothelial progenitor cells, with the fusion machinery and expressing the CRISPR system targeting one or more oncogenes may produce a syncytium where the oncogenes expression is abrogated, involving nearby cells. Such mechanism could then be stopped thanks to the inducible promoter control and the syncytium formation may fuel the inflammatory response, priming the action of the immune system. Currently such cancer cell-based treatments are studied as potential theranostic therapies [81] but if further developed, they might become also an interesting carrier for gene-editing therapies.

## Abbreviations

AAV: Adeno-associated virus
ArgNPs: Arginine gold nanoparticles
AV: Adenovirus
BER: Base Excision Repair
CAR: Chimeric antigen receptor
Cas: CRISPR associated
CPP: Cell penetrating peptide
CRISPR: Clustered regularly interspaced short palindromic repeats
DSBs: Double strand breaks
EMT: Epithelial to Mesenchymal Transition
GEMMs: Genetically engineered mouse models
gRNA: Guide RNA
HDR: Homologous Directed Repair
IDLVs: Integration-deficient lentiviral vectors
LHNPs: Liposome-templated hydrogel nanoparticles
LVs: Lentiviruses
NHEJ: Non Homologous End Join
NLS: Nuclear localization sequence
PAM: Protospacer-adjacent motif
RNP: Ribonucleoprotein
