# Publisher Correction to: delivery systems of CRISPR/Cas9-based cancer gene therapy

**DOI:** 10.1186/s13036-019-0169-0

**Published:** 2019-05-02

**Authors:** Alessio Biagioni, Anna Laurenzana, Francesca Margheri, Anastasia Chillà, Gabriella Fibbi, Mario Del Rosso

**Affiliations:** Department of Experimental and Clinical Biomedical Sciences, University of Florence, Viale G.B. Morgagni 50, 50134 Florence, Italy

**Correction to**: J Biol Eng (2018) 12: 33.

**https://doi.org/10.1186/s13036-018-0127-2**

In the original version of this article [1], published on 18 December 2018, there was 1 incorrect author name.

The incorrect author name was published as:

Mario so.

The correct author name is:

Mario Del Rosso.

The original publication of this article has been corrected.

The publisher apologizes to the authors and readers for the error and the delay in publishing this correction article.
